# Whole Cell Therapeutic Vaccine Modified With Hyper-IL6 for Combinational Treatment of Nonresected Advanced Melanoma

**DOI:** 10.1097/MD.0000000000000853

**Published:** 2015-05-29

**Authors:** Mackiewicz Jacek, Karczewska-Dzionk Aldona, Laciak Maria, Kapcinska Malgorzata, Wiznerowicz Maciej, Burzykowski Tomasz, Monika Zakowska, Rose-John Stefan, Mackiewicz Andrzej

**Affiliations:** From the Chair of Medical Biotechnology, University of Medical Sciences (MJ, LM, KM, WM, MZ, MA); Department of Diagnostics and Cancer Immunology, Greater Poland Cancer Centre (MJ, K-DA, WM, MA); Department of Medical and Experimental Oncology, Clinical Hospital of Poznan University of Medical Sciences, Poznan, Poland (MJ); Interuniversity Institute for Biostatistics and Statistical Bioinformatics, Hasselt University, Diepenbeek, Belgium (BT); Biochemisches Institut, University Kiel, Kiel, Germany (R-JS); and BioContract Sp z o.o., Poznan, Poland (LM, KM, MA).

## Abstract

Active specific immunotherapy of cancer requires an efficient induction and effector phase. The induction covers potent activation of anti-tumor response, whereas effector breaks the immunosuppression. We report efficacy of therapeutic melanoma vaccine (AGI-101H) used alone in advanced disease as a candidate for further combined treatment. In adjuvant setting in patients with resected metastases AGI-101H combined with surgery of recurring disease demonstrated long-term survival.

Seventy-seven patients with nonresectable melanoma (8% IIIB, 21% IIIC, 71% IV) were enrolled. AGI-101H was administered 8× every 2 weeks, and then every month. At progression, maintenance was continued or induction was repeated and followed by maintenance.

Median follow-up was 139.3 months. The median overall survival (OS) was 17.3 months; in patients with WHO 0-1 was 20.3 months. Complete response (CR) and partial response (PR) were observed in 19.4% and 9% of pts. Disease control rate was 54.5% of pts. The median CR+PR duration was 32 months. Reinduction was performed in 36.3% patients following disease progression with 46.6% of CR+PR. No grade 3/4 adverse events were observed.

Treatment with AGI-101H of melanoma patients is safe and effective. AGI-101H is a good candidate for combinatorial treatment with immune check-points inhibitors or tumor hypoxia normalizators.

Trial registration: EudraCT Number 2008–003373-40.

## INTRODUCTION

Active specific immunotherapy of cancer to be successful needs to generate efficient induction and effector phases of antitumor immune responses. The induction phase includes mounting of specific effector response, whereas the effector phase results in the eradication of the tumor. For a long time, it has been acknowledged and supported by model studies that tumor cells escape immune recognition, whereas the host requires proper cancer antigens presentation. Various approaches, which included therapeutic cancer vaccination, were tested in clinical trials, but they demonstrated only limited benefit for patients.^1,4,5^ Recent studies of the cancer-host immune interactions led to understanding of a role, which plays tumor-related local and systemic immune suppression in mounting effective cancer active specific immunotherapy.^6–8^ Identification of immune checkpoints and ways of their inhibition opened new perspectives for cancer active specific immunotherapy.^9^ Moreover, better understanding of local tumor immunosuppression driven by hypoxia and ways of hypoxia normalization to break the suppression may lead to further enhancement of cancer active specific immunotherapy clinical effectiveness.^10,11^

To date, no active specific immunotherapy including therapeutic cancer vaccines, peptides, DNA, dendritic cells (DCs) evaluated in phase III studies has shown extension of overall survival (OS) of patients with advanced melanoma.^1–5^ Improvement of OS of patients with castration-resistant advanced prostate cancer treated with Sipuleucel-T (Provenge, Dendreon, Seattle, WA), autologous DC vaccine loaded with prostate acid phosphatase fused with GM-SCF (Granulocyte-Macrophage Colony-Stimulating Factor), however, led to its marketing authorization. Results of the above phase III study demonstrated that therapeutic vaccination even as mono-therapy in well-designed clinical setting might be effective in cancer patients.^12^

Inhibitors of immune check-points such as ipilimumab (Yervoy, Bristol-Myers Squibb, New York) (antibody against cytotoxic T-lymphocyte-associated antigen 4 [CTLA-4]), or anti PD-1 (antibody against programmed death 1 protein)^13,14^ and anti-PD-1 ligand^15^ administered as mono-therapy demonstrated significant tumor reduction and extension of survival of melanoma patients. Preclinical studies of inositol triphosphate (ITPP)–hypoxia normalization agent showed reduction of melanoma and breast cancer tumors up to complete eradication.^16^ Breaking cancer related immunosuppression to bring the benefit to patients and eventually cure the disease, however, requires specific immune effector cells.^9^ Accordingly, therapeutic vaccination combined with inhibition of immune checkpoints or hypoxia normalization would be become necessary,^9–11,17–20^ Certainly, the vaccine is expected to mount specific immunological anti-melanoma responses and preferably tumor responses.

AGI-101H is a therapeutic melanoma gene-modified allogeneic cellular vaccine, which in adjuvant setting in combination with surgery of recurrent disease led to a significant long-term OS of advanced melanoma patients (stages IIIB–IV).^21^ Accordingly, AGI-101H may prove to become a good candidate for combinational treatment of advanced melanoma patients with measurable (nonresected) disease.

Here, we report results of a phase II trial conducted in 77 patients with metastatic stage III and IV melanoma immunized with AGI-101H.

## METHODS

Single-arm, prospective, open-label, single-institution, clinical study – “Trial 2” (“A phase II trial: the evaluation of the efficacy and toxicity of an allogeneic melanoma vaccine, genetically modified, with interleukin 6/soluble interleukin 6 receptor complex (Hyper IL-6) in patients with measurable melanoma metastases”) was carried out. The aim was to determine the efficacy and toxicity of a specific treatment based on the Hyper-IL6 (H6) gene modified whole-cell allogeneic melanoma vaccine. Patients with stage III or IV measurable melanoma who signed informed consent were eligible.

Inclusion criteria included: first, histologically proven malignant melanoma (stage III or IV) with all measurable metastases; second, previous chemotherapy or immunotherapy completed at least 4 weeks before enrolment; third, WHO Performance Status 0-2; fourth, men and women, age >18 years; fifth, informed consent signed before patient's enrolment; sixth, adequate haematology, liver, and renal tests; and seventh, male and female patients with reproductive potential had to use an approved contraceptive method during the study. There were deviations from the protocol, because 9 patients displayed WHO Performance Status 3-4.

The primary end point was OS; the secondary end points were toxicity, immune responses to the melanoma vaccine, laboratory predictors of the objective clinical response to the vaccine, and objective clinical responses.

Trial 2 was approved by the Regional Bioethics Committee (RBC) in Poznan, Poland (Decision No. 477A/97). In November 2008, all living patients from the study were transferred into the “Extended Treatment for Advanced Melanoma Patients Transferring From Trials 2-5 (ETAM 2-5)” trial. The objectives of the ETAM study are the long-term toxicity and outcome. All the patients signed informed consent. The ETAM trial was approved by the RBC and the Central Evidence of Clinical Trials (EnduraCT Number 2008-003373-40).

Inclusion criteria in the ETAM study were as follows: the patient had to participate in one of the previous studies conducted by the same Sponsor (Trials 2-5); the patient had to provide the signed informed consent for the participation in the study before any study-related procedures.

Patients with asymptomatic brain metastases were eligible to all presented studies.

### Vaccine Composition and Treatment

Details of vaccine composition are described in [22]. Briefly, AGI-101H is composed of 2 allogeneic melanoma cell lines Mich-1 and Mich-2, modified with cDNA encoding molecular adjuvant Hyper-IL-6 (H6),^22,23^ which were mixed 1:1. H6 is a fusion protein composed of interleukin 6 (IL-6) and its agonistic soluble IL-6 α receptor. Mich-1H6 are HLA-A1,A2, Mich-2H6 are HLA-A3 positive and express MAGE-A, A1, A9, A12 (MAGE A3, A6 negative) BAGE, GAGE-1, -2, -8, GAGE-3, -4, -5, -6 -7, 7B, NY-ESO1, gp100, CTp11, PRAME, NA17A, TRP-1, TRP-2, Sox-10, SSX-1, HD-MM-05, -07, -21, -22, -25. LAGE-1 is expressed in Mich2-H6.^22^ Vaccine was injected at dose of 5 × 10^7^ cells 8 times in 2 weeks intervals (induction) and then every month (maintenance) until patient's death. At progression, further management was at the discretion of the clinician. If disease progression was observed, reinduction followed by maintenance was permitted. If clinically indicated, palliative radiotherapy during vaccine treatment was permitted.

### Clinical Tumor Responses and Toxicity Assessments

Tumor responses following enrollment or reinduction were assessed by Response Evaluation Criteria in Solid Tumors (RECIST) criteria version 1.0 (complete response (CR) – the disappearance of all target lesions; partial response (PR) – at least a 30% decrease in the sum of the longest diameter of target lesions, taking as reference the baseline sum longest diameter; progressive disease – at least a 20% increase in the sum of the longest diameter of target lesions or the appearance of 1 or more new lesions; stable disease (SD) – neither sufficient shrinkage to qualify for partial response nor sufficient increase to qualify for progressive disease (PD)).^24^ Moreover, a term, disease control (DC), to express overall response rate was introduced. It reflects sum of patients with CR + PR + SD. The first in-study tumor assessment was performed after fourth vaccination (day 43). The second response evaluation was done after completion of induction phase (after next 12 weeks) and then every 16 weeks. If clinically indicated (eg, suspected progression), additional examinations were carried out. Local toxicities at the injection site or systemic toxicities such as body temperature, pain of the regional lymph nodes, arthritic pain, and systemic allergic reactions were recorded by patients. Toxicities were graded by the World Health Organization (WHO) criteria.

### Statistical Analyses

The median OS of at least 12 ± 1 month (approx. 90% CI) justifies further evaluation of AGI-101 in a randomized phase III study. For the sufficient evaluation of efficacy, an overall accrual of maximum 80 patients was calculated. This number of patients will enable getting a median OS with approximately 90% CI within the above assumed limits. OS time was computed from the date of the first vaccination to death (complete observations) or the date of the last observation (censored observations). Survival status of all patients was updated on August 1, 2013. OS functions were estimated by the Kaplan–Meier method. Confidence intervals (CI) for survival probabilities were computed by using the log–log transformation of the estimates of the probabilities. Median OS were estimated based on the estimated survival functions.^25^ Confidence intervals for the median OS were estimated based on the upper and lower limits of the confidence intervals of survival probabilities. The median follow-up time was estimated by using the “reverse” Kaplan–Meier method, that is, by treating deaths as censored observations.

All computations were conducted by using STATA v.11 (StataCorp LP, College Station, TX). Results of statistical significance tests were assessed using the 5% significance level (2-sided).

### Patients’ Enrollment and Characteristics

Between 1997 and 2001, 77 nonselected patients were enrolled into the trial. In November 2008, 7 patients were transferred to the ETAM study. On August 1, 2013, the median follow-up was equal to 12.3 years (Table 1).

**TABLE 1:**
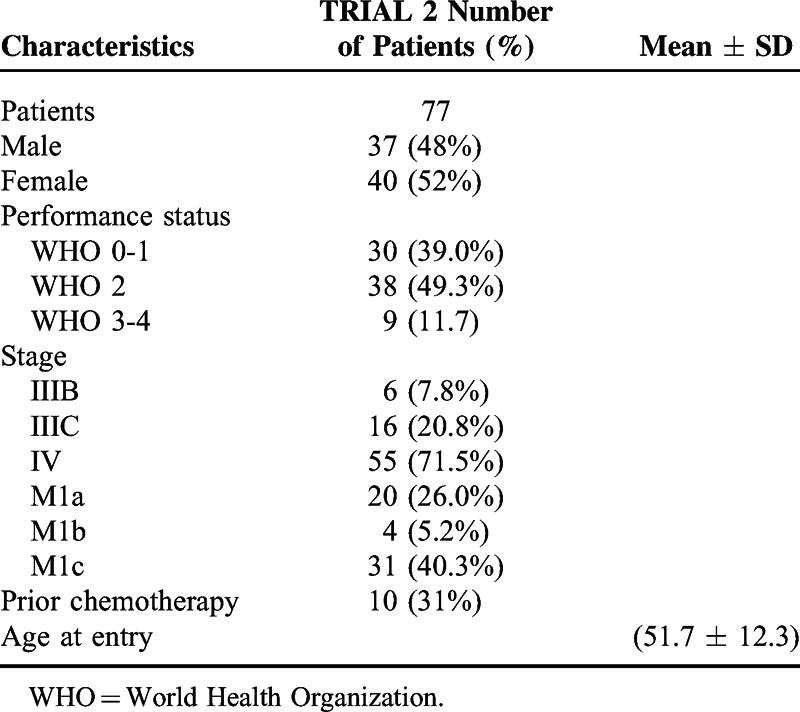
Patients Characteristics

## RESULTS

### Response Rate

Objective responses (complete response (CR), partial response (PR)) were observed in 22 (28.6%) patients, with CR and PR observed, respectively, in 15 (19.5%) and 7 (9.1%) patients. Disease control (DC=CR+PR+SD; SD – stabilization of the disease) was observed in 42 (54.5%) patients (Table 2).

**TABLE 2:**
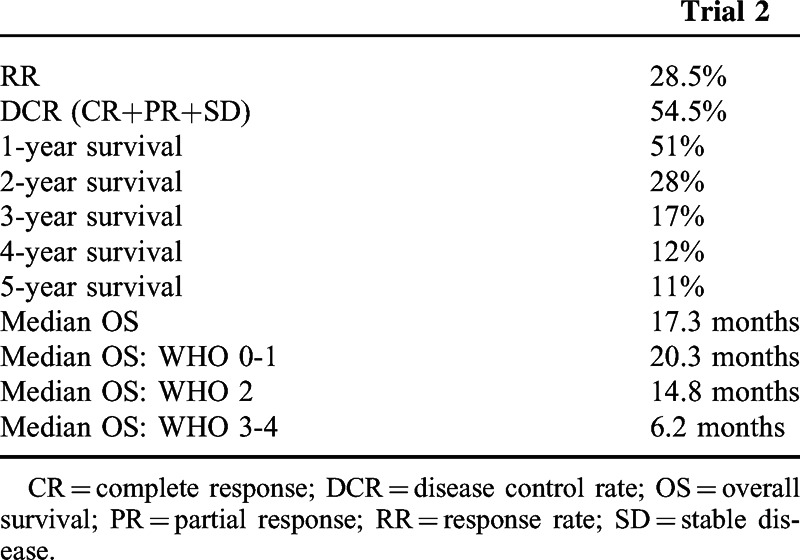
Response Rate, Progression-Free Survival, and Survival Observed in Trial 2

Patients developing CR and PR had median response duration of 32 months (95% CI: [6.1, 125.2]) and 6.3 months (95% CI: [3.0, 6.5]), respectively.

### Overall Survival

**FIGURE 1:**
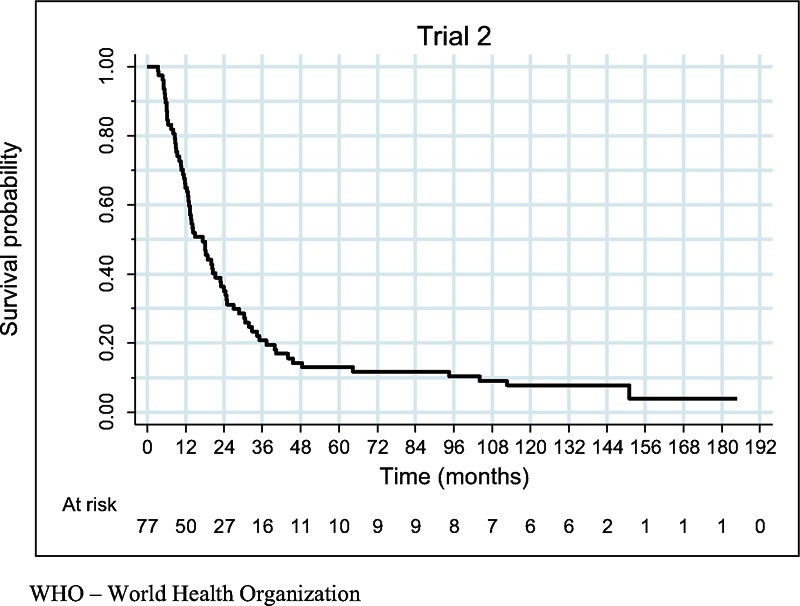
Overall survival function estimated by the Kaplan–Meier method.

**FIGURE 2:**
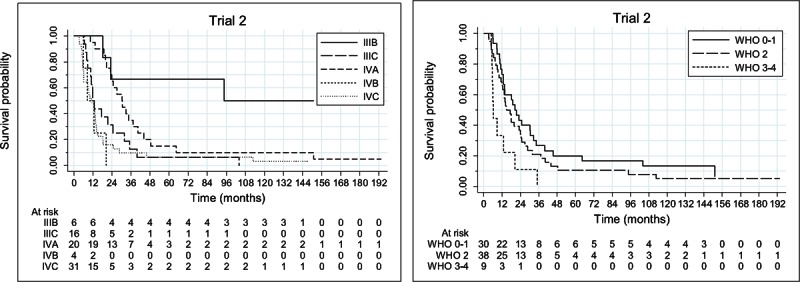
Overall survival function estimated by the Kaplan–Meier method per disease stage (left) and per World Health Organization performance status (right).

There were 72 deaths. All 5 (6.5%) remaining patients were alive at the data cut-off. All surviving patients achieved CR. The estimated survival probability at 1-, 2-, 3-, 4-, 5-year was equal to 64.9% (95% CI: [53.2%, 74.4%]), 35.1% (95% CI: [24.7%, 45.7%]), 20.8% (95% CI: [12.6%, 30.4%]), 14.3% (95% CI: [7.6%, 23.0%]), and 13% (95% CI: [6.7%, 21.5%]), respectively. The estimated median OS was equal to 17.3 months (95% CI: [12.9, 21.3]) (Figure 1). The estimated median OS of patients with stage IIIB was equal to 94.6 months; stage IIIC – 11.9 months; stage IV-M1a – 30.2 months; IV-M1b – 8.6 months; IV-M1c – 11.4 months (Figure 2). The estimated median OS of WHO 0-1, 2, and 3-4 performance status patients was equal to 20.4 (95% CI: [12.9, 32.7]), 14.8 (95% CI: [11.9, 23.8]), and 6.2 (95% CI: [5.1, 20.3]) months, respectively (Figure 2 and Table 2). The median OS of 5 (6.6%) patients with asymptomatic brain metastases was 20 months [95% CI: (8.5, upper bound unestimable)]. Four patients did not receive any local treatment due to brain metastases before entering the study. One patient was treated with whole brain radiotherapy.

### Reinduction

Reinduction therapy was performed in 28 (36.3%) patients after disease progression. Objective response rate was observed in 13 (46.4%) of those patients.

### Toxicity

No grade 3 or 4 toxicity was observed in the study. Expected toxicity was related to local vaccine reaction, manifested by redness, edema and itching at the injection site. Systemic toxicities such as fever, sweating, or myalgia were occasional and mild (grades 1, 2).

## DISCUSSION

There are 4 major findings of the study: therapeutic vaccination with AGI-101H of advanced melanoma patients with measurable disease resulted in durable clinical responses and prolonged median OS, application of vaccine reinduction regimen in progressing patients led to subsequent objective clinical responses, patients with WHO performance status 0-1 presented the longest median OS.

The aforementioned findings indicate that AGI-101H may prove a good candidate for combinatorial treatment with inhibitors of immune check-points or tumor hypoxia normalization agents.

Our parallel 2 phase 2 studies (median follow-up 10.5 and 6.2 years, respectively) in patients with advanced resected melanoma (stages IIIB–IV) demonstrated significant fraction of long-term survivors after treatment with AGI-101H. Patients treated in the adjuvant setting and receiving reinduction with AGI-101H after disease progression demonstrated up to 70% reduction of the risk of death compared with patients not reinduced.^21^

One/third of patients of the present study demonstrated objective responses (CR + PR) with substantial median duration, whereas DC was observed in 54% of patients. In contrast, results of the phase 3 study (MDX010–20) leading to the approval of ipilimumab (anti-CTLA4) for the treatment of metastatic melanoma demonstrated clinical response rate in 11% (CR – 1.5%; PR – 9.5%) of patients, with DC rate of 28.5%. The median duration of response was not estimable in patients treated with ipilimumab alone. In patients treated with ipilimumab and gp100, in which the response rate and OS data were similar to those observed in ipilimumab alone arm, the median duration of response was, however, 11.5 months.^26^ Other very promising immunomodulating agent – pembrolizumab (Keytruda, Merck, Kenilworth, NJ) (anti-PD1) was recently approved by the US Food and Drug Administration (FDA) for the treatment of advanced melanoma following ipilimumab and, if *BRAF V600* mutation positive, a BRAF inhibitor. Pembrolizumb was approved under accelerated approval based on tumor response rate (overall response rate – 24%) and duration of response presented in a phase 1 study conducted in 173 patients. At the data cutoff, 86% of patients had ongoing responses with durations ranging from 1.4+ to 8.5+ months.^27^ Nivolumab (Opdivo, Bristol-Myers Squibb), another anti-PD1 drug evaluated in a phase 1 study, showed objective responses in 34 (32%) of 107 metastatic melanoma patients, median response duration was 22.9 months. The 2- and 3-year OS rates were 48% and 41%, respectively.^14^ In a recently presented phase 3 studies in patients with advanced melanoma after prior anti-CTLA4 therapy, the objective response rate was also 32% in patients treated with nivolumab comparing to 11% in the chemotherapy arm. OS was not analyzed at the time of interim analysis.^13^

The latest results from phase III study demonstrated improvement in survival and PFS (progression-free survival) in patients treated with nivolumab comparing to dacarbazine (Dacarbazin TEVA, TEVA, Petah Tikva, Israel) in the first line treatment (1-year survival 73% vs 42%, HR = 0.42, *P* < 0.001).^28^ Also another immunomodulating antibody directed against PD-L1 (anti-PD-L1; programmed death ligand 1) was recently tested in a phase 1 study. Among patients who could be evaluated at data cut-off objective response was observed in 9 (17%) of 52 advanced melanoma patients. At all evaluated doses of anti-PD-L1 the duration of response was from 2.8 to at least 23.5 months.^15^

The estimated median OS in patients treated with AGI-101H was equal to 17.3 months. In patients with a WHO 0-1 performance status (PS), the estimated median OS was, however, equal to 20.3 months. In the phase 3 regulatory study, which enrolled mainly WHO 0-1 PS patients (98%), the estimated median OS in patients receiving ipilimumab was equal to 10.5 months.^26^ In the study evaluating nivolumab, the estimated median OS was equal to 16.8 months across all studied doses and 20.3 months at the 3 mg/kg dose selected for phase 3 trial. Due to slightly different patients characteristics, all these studies are, however, not directly comparable. Various immunotherapeutic agents such as interleukin-2 (IL-2), ipilimumab, nivolumab, or anti-PD-1L demonstrate a long-lasting survival of fraction of patients, which can eventually translate into the cure of metastatic melanoma. In our study, the estimated 5-year survival probability was equal to 11%. Moreover, 6.6% of patients were alive at data cutoff. Furthermore, all surviving patients treated with AGI-101H developed CR.

Treatment with AGI-101H is safe. Through all AGI-101H trials conducted in over 400 patients in the adjuvant and measurable disease settings, only grade 1 and 2 toxicities were observed. In contrast, other immunotherapy strategies such as IL-2, interferon-alfa, ipilimumab, nivolumab, or anty-PD-1L were linked with high rate of grade 3 or 4 adverse events, which might be life-threatening.

The beneficial effects of reinduction with immunotherapeutic agents were reported.^21,26,29^ In our study, 31% of progressing patients received reinduction followed by maintenance phase. Clinical responses to the reinduction were observed in 46% of patients vaccinated with AGI-101H. These data and results of adjuvant studies show that in patients with disease progression (excluding massive or symptomatic progression) treatment with AGI-101H should be continued, whereas approximately half of the patients benefit from the reinduction.^21^

In preclinical models, it has been observed that anti-CTLA4 and anti-PD1 therapy strongly enhances the amplitude of vaccine-induced antitumor response.^30,31^ It has been described that immune checkpoint ligands such as PD-L1 are upregulated in tumors in response to endogenous antitumor immune response. This observation suggests that blocking the PD-1 pathway will eradicate tumors only with the preexisting endogenous antitumor immune response. Furthermore, activation of antitumor immune response by cancer vaccines may not induce tumor regression, whereas tumors respond by upregulating immune checkpoint ligands inducing immunosuppression.^9^ Very encouraging results coming from early phase trials have been presented in patients treated with cancer vaccines combined with immune checkpoint inhibitors such as anti-PD1^9,20,32^ and anti-CTLA4.^17,18,33^ In our study, each AGI-101H dose administration induced increase of the number of melanoma antigen-specific CD8+ cytotoxic lymphocytes (CTL) (data not published). These CTL expressed PD-1 receptor thus became exposed and susceptible to neutralization by melanoma cells (of the tumor) expressing PD-L1. Accordingly, addition of anti-PD-1 blocking antibodies to the vaccine therapy would preserve specific CTL effector cells.

## CONCLUSIONS

Presented results taken together with results of our studies in advanced melanoma patients with resected metastases indicate that therapeutic vaccination with AGI-101H may prove a perfect candidate for combinational treatment with anti-PD-1, anti-PD-1L, or tumor hypoxia normalization agents.^9,10,11,21^ The above conclusion is strongly supported by our previous study in which AGI-101H therapeutic vaccination was combined with surgery of recurring metastases during treatment which resulted in long-term survival of the patients.^21^ In this setting, surgical removal of tumors “mimicked” blockade of tumor-induced immunosuppression by a-PD-1, a-PD-1L, or antihypoxia agents. Moreover, AGI-101H does not require HLA matching as for ex. peptide vaccines do.
