# Whole Cell Therapeutic Vaccine Modified With Hyper-IL6 for Combinational Treatment of Nonresected Advanced Melanoma: Erratum

**DOI:** 10.1097/01.md.0000467316.91878.b5

**Published:** 2015-06-26

In the article “Whole Cell Therapeutic Vaccine Modified With Hyper-IL6 for Combinational Treatment of Nonresected Advanced Melanoma”,^1^ which appeared in Volume 94, Issue 21 of *Medicine*, the authors’ first and last names were incorrectly transposed in the byline. The article has since been corrected online.

## References

[1] Jacek M Aldona K-D Maria L Whole cell therapeutic vaccine modified with hyper-IL6 for combinational treatment of nonresected advanced melanoma. *Medicine* 94 21:e853. 26020391 10.1097/MD.0000000000000853 PMC4616404

